# Acetylated KHSRP impairs DNA‐damage‐response‐related mRNA decay and facilitates prostate cancer tumorigenesis

**DOI:** 10.1002/1878-0261.13634

**Published:** 2024-03-19

**Authors:** Haihua Yuan, Renjie Cai, Biying Chen, Qian Wang, Mengting Wang, Junyi An, Weishu An, Ye Tao, Jianxiu Yu, Bin Jiang, Yanjie Zhang, Ming Xu

**Affiliations:** ^1^ Department of Oncology, Shanghai Ninth People's Hospital Shanghai Jiao Tong University School of Medicine China; ^2^ Shanghai Institute of Precision Medicine, Shanghai Ninth People's Hospital Shanghai Jiao Tong University School of Medicine China; ^3^ Department of Biochemistry and Molecular Cell Biology, Shanghai Key Laboratory of Tumor Microenvironment and Inflammation Shanghai Jiao Tong University School of Medicine China

**Keywords:** Acetylation, DNA damage response, KHSRP, prostate cancer, tumorigenesis

## Abstract

Androgen‐regulated DNA damage response (DDR) is one of the essential mechanisms in prostate cancer (PCa), a hormone‐sensitive disease. The heterogeneous nuclear ribonucleoprotein K (hnRNPK)‐homology splicing regulatory protein known as far upstream element‐binding protein 2 (KHSRP) is an RNA‐binding protein that can attach to AU‐rich elements in the 3′ untranslated region (3′‐UTR) of messenger RNAs (mRNAs) to mediate mRNA decay and emerges as a critical regulator in the DDR to preserve genome integrity. Nevertheless, how KHSRP responds to androgen‐regulated DDR in PCa development remains unclear. This study found that androgen can significantly induce acetylation of KHSRP, which intrinsically drives tumor growth in xenografted mice. Moreover, enhanced KHSRP acetylation upon androgen stimuli impedes KHSRP‐regulated DDR gene expression, as seen by analyzing RNA sequencing (RNA‐seq) and Gene Set Enrichment Analysis (GSEA) datasets. Additionally, NAD‐dependent protein deacetylase sirtuin‐7 (SIRT7) is a promising deacetylase of KHSRP, and androgen stimuli impairs its interaction with KHSRP to sustain the increased KHSRP acetylation level in PCa. We first report the acetylation of KHSRP induced by androgen, which interrupts the KHSRP‐regulated mRNA decay of the DDR‐related genes to promote the tumorigenesis of PCa. This study provides insight into KHSRP biology and potential therapeutic strategies for PCa treatment, particularly that of castration‐resistant PCa.

## Graphical abstract

KHSRP deacetylated by SIRT7 feasibly decays mRNAs of DNA damage response (DDR) genes to weaken DNA repair ability, resulting in prostate cancer (PCa) cell death. KHSRP–SIRT7 interaction is attenuated upon androgen receptor (AR) activation in PCa cells, which leads to acetylated KHSRP inefficiently reaching the adenine uridine (AU)‐rich elements (AREs) in the 3′‐UTR of DDR genes to exert mRNA decay function.

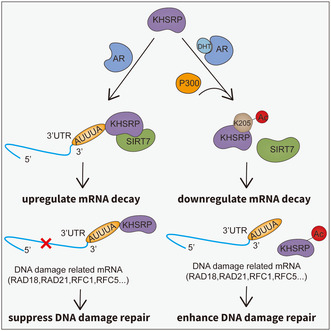

**Abbreviations**
3′‐UTR: 3′‐untranslated region
ADT: androgen deprivation therapy
AR: androgen receptor
AREs: adenine uridine (AU)‐rich elements
ATM: Ataxia‐telangiectasia mutated kinase
BRCA: breast cancer susceptibility gene
DDR: DNA damage response
DHT: dihydrotestosterone
GSEA: Gene Set Enrichment Analysis
IHC: immunohistochemistry
KHSRP: hnRNP K homology (KH)‐type splicing regulatory protein
mRNAs: messenger RNAs
NAM: nicotinamide
PARP: poly(ADP‐ribose) polymerase
PCa: prostate cancer
PTMs: post‐translational modifications
RBPs: RNA‐binding proteins
RNA‐seq: RNA sequencing
SIRT7: sirtuin‐7
SRB: Sulforhodamine B
TL: terminal loop
TSA: trichostatin A

## 1 Introduction

Prostate cancer (PCa) is the second diagnosed malignant tumor in men and the sixth in mortality in all tumors. Finding more effective means to diagnose and treat PCa has been urgent and essential [1]. Androgen receptor (AR) plays a significant role in the growth and progression of PCa in all stages and is a crucial therapeutic target in clinical [2, 3]. About 80~90% of PCa remains sensitive to androgen at the initial diagnosis, and androgen deprivation therapy (ADT), which aims to reduce serum androgen and inhibit the function of androgen receptors, is currently administered as primary systemic standard treatment for regional or advanced PCa [4, 5]. In the early stage of therapy, ADT can often effectively inhibit the growth of PCa and delay the progression of the disease. However, with the duration of anti‐androgen therapy extending, the AR gradually obtains androgen‐independent activation, resulting in PCa from castration‐sensitive to castration‐resistant [6, 7, 8], making patients face an increased risk of metastasis and eventual death [9].

In castration‐resistant PCa, AR is continuously activated to drive tumor progression. It remains clinically valuable to dig therapeutic targets in the AR signaling pathway. Recently, a large subset of DDR gene transcripts in PCa has been reported and functions in enhancing the capacity of DNA damage repair and promoting radioresistance [10, 11], which opened up new horizons for castration‐resistant PCa treatment. For example, the FDA‐approved Olaparib, an inhibitor of pan‐Poly(ADP‐ribose) Polymerase (PARP) in the DDR pathway, has been clinically used for treating Breast Cancer Gene 1/2 (BRCA1/2)‐deficient PCa [12], making research significant on AR downstream signaling, particularly in the DDR pathway.

The KH‐type splicing regulatory protein, KHSRP, was reportedly targeted for phosphorylation by ATM in response to DNA damage, which is essential for pri‐miRNA processing [13, 14]. Being an AU‐RBP with a central region of four KH domains (KH1‐4), KHSRP comprises two main functions: miRNA maturation and mRNA decay [15]. In the miRNA processing progress, KHSRP is a critical component of either the Drosha complex that cleaves pri‐miRNAs into pre‐miRNAs featured with stem‐loop structure in the nuclear or the Dicer complex that processes pre‐miRNAs into mature miRNA duplexes in the cytoplasm through binding to the terminal loop of regulated miRNAs [16]. Within all the functional sequences in the 3′‐UTR of mRNA, AREs characterized by AUUUA pentamer in the AU context have been widely known for the mRNA decay function [17]. Once KHSRP is combined with the AREs of target mRNAs, the third and fourth KH domains of KHSRP quickly start to complex with mRNA decay enzymes, including the poly (A) RNase PARN, the exosome components, and the decapping enzyme DCP2, to realize its mRNA decay function [18, 19, 20]. Significantly, the KHSRP function can be influenced by various critical post‐translational modifications (PTMs) [13, 21, 22]. For instance, KHSRP phosphorylation mediated by MAPK/p38 affects the interaction between KHSRP and the AREs of target transcripts and inhibits its promotion of myogenic mRNA degradation [23]. While KHSRP SUMOylation can suppress PCa growth by preventing TL‐G‐Rich miRNA biogenesis [22]. Despite this, whether KHSRP‐regulated mRNA decay in response to androgen stimuli or AR activity in the PCa process is still in the puzzle.

Here, we report that KHSRP acetylation, a new post‐translational modification of KHSRP, is tightly associated with AR activity and DDR regulation, which serves tumor growth and malignancy by impairing DDR‐related mRNA decay in PCa. The biological relevancy between KHSRP acetylation and DDR was addressed further to understand the AR‐regulated DDR in this cancer disease.

## 2 Materials and methods

### 2.1 Cell culture and cell lines

Human embryonic kidney 293T (HEK‐293T, RRID: CVCL_0063), LNCaP (RRID: CVCL_0395), 22RV1 (RRID: CVCL_1045), and DU145 (RRID: CVCL_0105) cell lines were purchased from the Cell Bank of Type Culture Collection of the Chinese Academy of Sciences (Shanghai, China). LNCaP and 22RV1 cell lines were cultured in Roswell Park Memorial Institute (RPMI) 1640 medium (Hyclone, Logan, UT, USA) with 10% fetal bovine serum (BioSun, Shanghai, China), 100 mg·mL^−1^ streptomycin, 100 U·mL^−1^ penicillin (Hyclone), and 1% GlutaMAX (Gibco, Grand Island, NY, USA). HEK‐293T and DU145 cell lines were cultured in high‐glucose Dulbecco's Modified Eagle Medium (DMEM) (Hyclone) with the abovementioned supplements. All cell lines were cultured at 37 °C in a 5% CO_2_ humidified incubator. Cells were authenticated by STR profiling and tested for mycoplasma contamination.

### 2.2 Antibodies and reagents

ACE‐lysine antibody (ab21623), anti‐KHSRP antibody (ab150393), anti‐SIRT7 antibody (ab259968), anti‐γH2AX antibody (ab81299), anti‐Ki67 antibody (ab15580), and anti‐Histone H3 (acetyl K18) antibody (ab40888) were purchased from Abcam (Cambridge, MA, USA). Antibodies against mouse M2 Flag‐tag antibody (F1804), dihydrotestosterone (A8380‐1G), and Actinomycin D(A9415) were purchased from Sigma‐Aldrich (St. Louis, MO, USA), Trichostatin A (TSA) (#S1045), Nicotinamide (NAM) (#S1899), and protease inhibitor cocktail (EDTA Free, 100× in DMSO), ARN‐509 (#S2840) were purchased from Selleck (Houston, TX, USA). Pierce™ Protein A/G magnetic beads were purchased from ThermoFisher Scientific (Waltham, MA, USA). HA‐Tag Rabbit (C29F4, #3724) was purchased from Cell Signaling Technology (Danvers, MA, USA). IgG fraction monoclonal mouse anti‐rabbit IgG (light chain‐specific) was purchased from Jackson ImmunoResearch (West Grove, PA, USA). Etoposide (HY‐13629) was purchased from MedChemExpress (Monmouth Junction, NJ, USA).

### 2.3 Plasmids, site‐directed mutagenesis, transfection, and lentivirus infection

The construction of HA‐KHSRP and Flag‐KHSRP plasmid has been described previously [22]. Mutations of KHSRP were generated using the KOD‐plus‐mutagenesis kit (TOYOBO, Osaka, Japan) and the Exnase II enzyme system (Vazyme, Nanjing, China) according to the manufacturer's instructions. Cell transfection was performed using Hiff‐Trans™ Liposomal Transfection Reagent (YEASEN, Shanghai, China). To silence the endogenous KHSRP in LNCaP cells and re‐applied HA‐tagged KHSRP in LNCaP cells, we used the homemade lentiviral vector pGreenPuro‐Dual for the recombinant constructs, which can silence endogenous genes and express exogenous genes with a single lentiviral infection as described previously [24]. All the primers are listed in Table S1.

### 2.4 Tissue microarray IHC

IHC was performed on the human PCa tissue microarrays obtained from Shanghai Outdo Biotech Co., Ltd (Shanghai, China). Samples were stained with an anti‐KHSRP antibody and homemade KHSRP‐acetyl‐K205 specific antibody overnight at 4 °C. Subsequently, the secondary antibodies conjugated with horseradish peroxidase were used for 1 h incubation at 37 °C. Finally, a total of 90 pairs of cancer and non‐cancer tissue samples were stained, imaged, and then scored by three independent pathologists. After removing incomplete or exfoliated samples, 69 pairs of samples were retained and included in statistics. IHC data are blindly analyzed and scored by three independent pathologists. Staining intensity: 0 = no staining, 1 = weak staining, 2 = moderate staining, 3 = strong staining; the frequency of positive cells: 0 = < 10%, 1 = 10–25%, 2 = 25–50%, 3 = 50–75%, 4 = > 75%. The final IHC score was summed with the staining intensity times the frequency of positive cells.

### 2.5 Nuclear/cytosol fractionation assay

The Nuclear/Cytosol fractionation kit (Beyotime Biotechnology, Shanghai, China) was used to extract nuclear and cytosolic fractions according to the protocol. Briefly, a total of 3 × 10^6^ cells were harvested for Nuclear/Cytosol fractionation and subjected to immunoprecipitation and western blotting assays with the indicated antibodies.

### 2.6 Immunoprecipitation

Cells were harvested and lysed with the RIPA lysis buffer (150 mm NaCl, 50 mm Tris–HCL, pH 7.4, 1% NP‐40, 0.01% SDS, and a complete protease inhibitor cocktail). Cell lysates were incubated at 4 °C overnight with Pierce Protein A/G mix magnetic beads and specific antibodies. After washing with the lysis buffer three times, the immunoprecipitants were boiled for SDS/PAGE resolving and immunoblotting.

### 2.7 Acetylation assays

HEK‐293T cells were transfected with HA‐tagged KHSRP and treated with 2 μm TSA for 16 h and 5 mm NAM for 2 h before harvesting. Cell lysates in RIPA lysis buffer (150 mm NaCl, 50 mm Tris–HCL, pH 7.4, 1% NP‐40, and a complete protease inhibitor cocktail) were incubated with magnetic beads and specific antibodies at 4 °C overnight, followed by immunoblotting to determine the acetylation level of KHSRP.

### 2.8 Cell proliferation assay

The SRB colorimetric assay was used to determine cell proliferation. Briefly, cells were seeded in quintuplicate into 96‐well plates (3000 cells per well). Cells were fixed with 100 μL 25% trichloroacetic acid (Sigma, T6399) at 4 °C for 1 h. After rinsing the plate with pure water and air drying, cells were stained with 100 μL of 0.05% SRB (S1402, Sigma‐Aldrich) in 1% acetic acid for 30 min. The plates were washed with 1% acetic acid to remove the unbound dye. The cells were dried before being added with 200 μL of 10 mm Tris base solution (pH 10.5) to solubilize the dye. Finally, the absorbance was measured at 510 nm to quantify the cell viability.

### 2.9 Animal xenograft

Twenty‐four 4‐week‐old male NOD‐SCID mice (NOD C.B7‐Prkdcscid/NcrCrl) were purchased from Weitong Lihua Laboratory Animal Technology (Beijing, China) and were kept in an isolated SPF (specific pathogen‐free) environment with temperature (25~26 °C), humidity (60~70%), ammonia concentration (less than 14 mg·m^−3^), light intensity (15~20 lux), noise (less than 60 dB), air change (10~15 times·h^−1^), and 12 h day‐night cycle. The mice were randomly divided into four required groups with similar weight, size, and healthy state, and at least five mice in each group were guaranteed after potential attritions. 100 μL of PBS containing 1 × 10^7^ indicated cells were mixed with an equal amount of Matrigel (Corning, 354248, Corning, NY, USA) for subcutaneous injection. Mice were sacrificed at 4 weeks post‐injection, and the tumor size and weight were measured to analyze and generate the curve chart. Mice that died or were injured due to biting were excluded from the statistical analysis; however, no mouse died or was injured in this animal trial. All animal operations including breeding, welfare, execution, and protocols, were conducted with the approval and guidance of the Ethical Animal Care and Use Committee of Shanghai Jiao Tong University School of Medicine Affiliated Shanghai Ninth People's Hospital (Document No. HKDL[2018]217).

### 2.10 RNA sequencing and data analysis

The total RNA was extracted using the VAHTS Universal V6 RNA seq Library Prep Kit (Vazyme, NR604‐02) for Illumina to construct the cDNA library. The complementary DNA library was sequenced using Illumina Nova seq6000 with 2 × 150 running circles. The unique original Fastq readings were mapped to the human transcriptome using the STAR procedure. The human transcriptome annotation file was retrieved and downloaded from the ensembl genome browser 111 (GRCh38.p14, Cambridge, Cambridgeshire, UK). The human genome was retrieved and downloaded from the ucsc Genome Browser (version hg38, Santa Cruz, CA, USA). Only genes with readings were analyzed using deseq2 to screen differentially expressed genes. The differential transcripts between the two groups were filtered for comparing the treated samples with the control samples according to the differential expression range satisfying |log_2_Fc| ≥ 0.2 and *P*‐value ≤ 0.05. The RNA‐seq raw data were deposited into the NCBI database (GEO number: GSE206486).

### 2.11 qRT‐PCR

Total RNA was extracted from the LNCaP stable cell lines using TRIzol (Invitrogen, Carlsbad, CA, USA). One microgram of total RNA was reverse‐transcribed using a PrimeScript™ RT reagent Kit with gDNA Eraser (Takara, Kusatsu, Shiga, Japan). RT‐qPCR was performed using SYBR Green (Takara). Primers are listed in Table S1. The gene expression levels of target genes were normalized to that of GAPDH.

### 2.12 Statistical analysis

Statistical analysis was performed using graphpad prism 8 (GraphPad Software, San Diego, CA, USA). Representative data are shown as the Mean ± SD from at least three independent experiments for SRB assay, 3D cell culture, animal xenograft, and qPCR. Paired or unpaired *t*‐test was used for the significance analysis. *P* < 0.05 was considered as statistically significant (*), < 0.01 very significant (**), or < 0.001 extremely significant (***). Statistical analysis Each presented cell experiment was set in duplicate or triplicate and performed at least three times for the power analysis.

## 3 Results

### 3.1 KHSRP can be acetylated mainly at the lysine 205

**Fig. 1:**
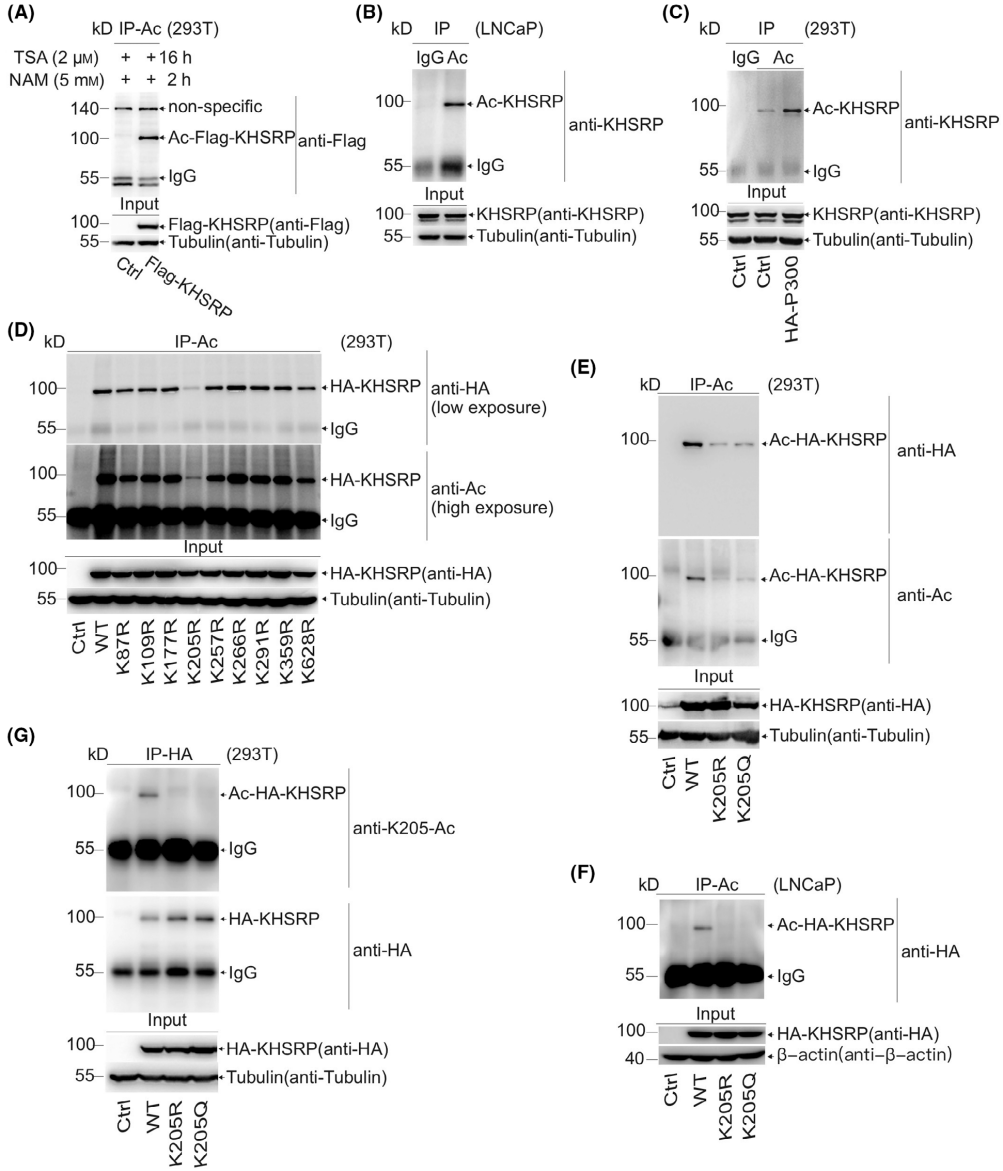
KHSRP can be acetylated mainly at K205. (A) HEK‐293T cells transfected with indicated plasmids were harvested after treating with 2 μm trichostatin (TSA) and 5 mm nicotinamide (NAM) for 2 and 16 h, and cell lysates in RIPA buffer were immunoprecipitated by the anti‐Acetylation (anti‐Ac) antibody. KHSRP acetylation level was analyzed by immunoblotting with indicated antibodies. (B) Lysates of LNCaP cells were immunoprecipitated with anti‐IgG or anti‐Ac antibodies, and KHSRP acetylation levels were analyzed by immunoblotting. (C) HEK‐293T cells transfected with indicated plasmids were lysed and immunoprecipitated with indicated antibodies. Endogenous KHSRP acetylation levels were determined by immunoblotting. (D) HEK‐293T cells were transfected with indicated plasmids and lysed for immunoprecipitation with the anti‐Ac antibody. The acetylation levels were detected by immunoblotting. (E–G) HA‐tagged KHSRP‐WT, ‐K205R, or ‐K205Q were transiently transfected into HEK‐293T cells (E, G), and HA‐KHSRP‐WT, K205R, or K205Q were re‐applied in KHSRP‐silenced LNCaP cells (F). The cell lysates in the RIPA buffer were immunoprecipitated with indicated antibodies. KHSRP acetylation levels were determined by anti‐Hemagglutinin (anti‐HA) (E, F), anti‐Acetylation (anti‐Ac) (E), or homemade anti‐K205‐Ac antibodies (G). All the experiments were replicated three times.

To detect whether KHSRP can be acetylated, Flag‐tagged KHSRP was transfected into the HEK‐293T cells. The acetylation of exogenous and endogenous KHSRP was analyzed and distinguishably observed by the immunoprecipitation assay (Fig. 1A,B). KHSRP acetylation was enhanced following the expression of a general acetylase p300 (Fig. 1C). Mass spectrometric analysis of the immunoprecipitated HA‐tagged KHSRP confirmed that nine lysine (K) residues in KHSRP were acetylated including K‐87, ‐109, ‐177, ‐205, ‐257, ‐266, ‐291, ‐354, and ‐628 (Fig. S1). Next, a series of point mutations of K to arginine (R) revealed that K205 significantly reduced the KHSRP acetylation level according to the immunoprecipitation assay (Fig. 1D). Moreover, HEK‐293T cells or LNCaP cells harboring HA‐KHSRP‐WT, ‐K205R, or ‐K205Q were extracted for immunoprecipitation, respectively. Western blotting analysis showed that either K205R or K205Q mutation contributed to noticeable reductions in KHSRP acetylation (Fig. 1E,F).

To further confirm the acetylation of KHSRP, we manufactured an antibody specifically against acetylated K205. A dot‐blot assay verified the specificity of the antibody, and we found that the KHSRP‐acetyl‐K205 antibody preferentially detected the acetylated but not the unmodified peptide (Fig. S2). Differential acetylation levels between ectopically expressed HA‐KHSRP‐WT and ‐K205R in HEK‐293T cells were also successfully detected by the KHSRP‐acetyl‐K205 antibody (Fig. 1G). Together, these results demonstrated that KHSRP is majorly acetylated at K205, and the KHSRP‐acetyl‐K205 antibody can be used in further analysis.

### 3.2 SIRT7 is a specific deacetylase of KHSRP

**Fig. 2:**
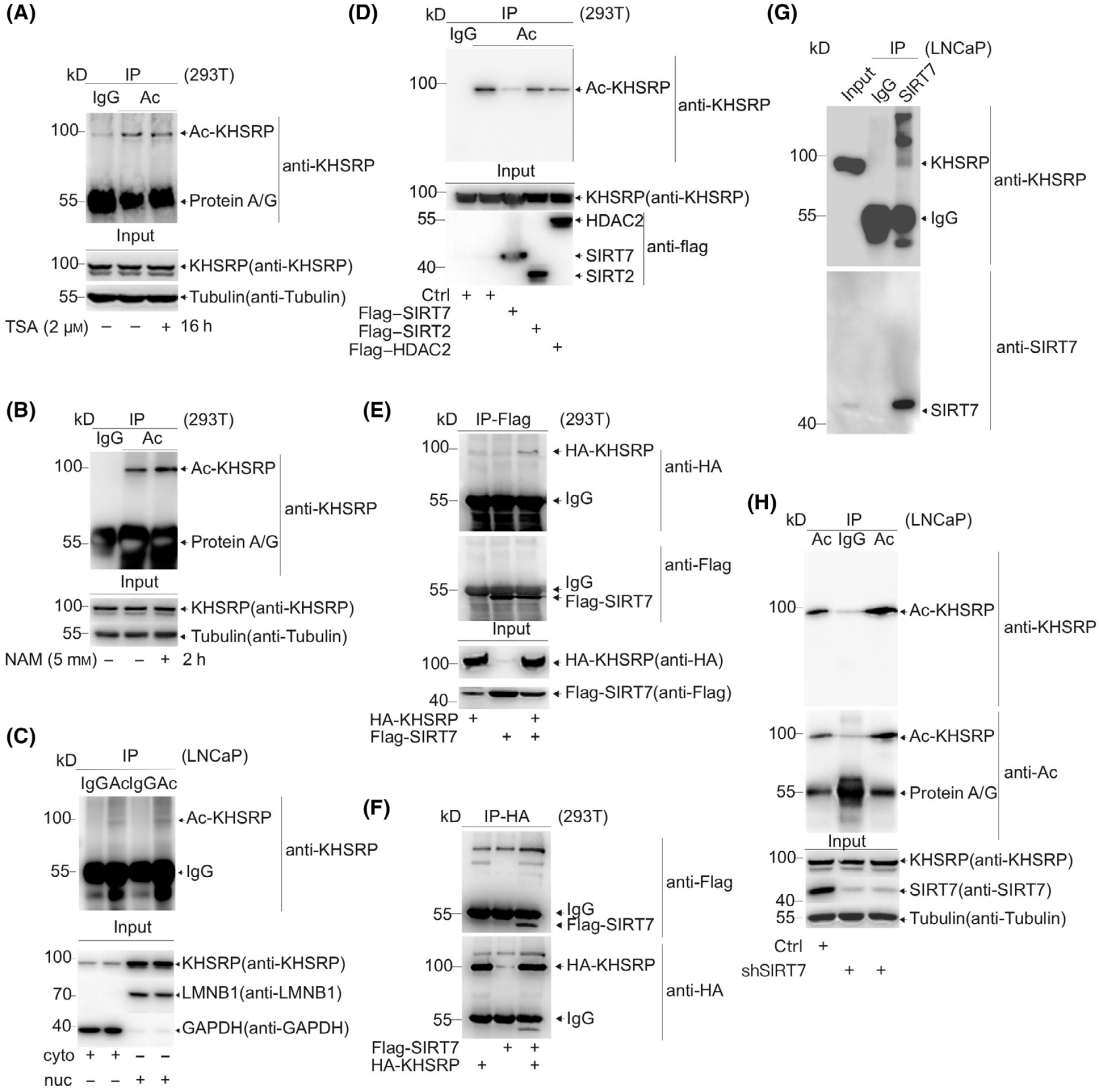
SIRT7 is the specific deacetylase of KHSRP. (A, B) HEK‐293T cells treated with trichostatin (TSA) for 2 h (A) or 5 mm nicotinamide (NAM) for 16 h (B) were harvested and lysed for immunoblotting to analyze the acetylation levels. (C) LNCaP cells were lysed and subjected to nuclear and cytoplasmic extraction, and then the acetylation of KHSRP was analyzed by immunoblotting. (D) Flag‐tagged HDAC2, SIRT2, or SIRT7 were transfected into HEK‐293T cells, respectively. The acetylation levels of KHSRP were analyzed by immunoprecipitation and immunoblotting. (E–G) The lysates from HEK‐293T co‐transfected with HA‐tagged KHSRP and Flag‐tagged SIRT7 (E, F) or LNCaP harboring Flag‐SIRT7 by lentiviral infection (G) were immunoprecipitated with indicated antibodies. The protein interactions were determined by immunoblotting. (H) The SIRT7 gene transcript was silenced through the lentiviral shRNA system in LNCaP, and the endogenous KHSRP acetylation level was determined by immunoprecipitation and immunoblotting with indicated antibodies. The presented results are representative of findings obtained from three independently replicates.

Since p300 can acetylate KHSRP, we wonder which deacetylase responds to removing the acetyl group in KHSRP. To identify the deacetylase of KHSRP, we treated HEK‐293T cells with the HDAC family inhibitor trichostatin A (TSA) or the Sirtuin family inhibitor NAM, respectively. We found that NAM but not TSA could enhance the KHSRP acetylation level (Fig. 2A,B), suggesting that Sirtuin family members are responsible for KHSRP deacetylation. To determine the subcellular distribution of acetylated KHSRP, we separated the LNCaP cell lysis into cytosolic and nucleoplasmic fractions, and further analysis indicated that KHSRP acetylation both occurs in the cytoplasm and nucleus (Fig. 2C). Considering that KHSRP expression in the cytoplasm is much lower than that in the nucleus, a higher proportion of KHSRP is acetylated in the cytoplasm and may perform essential functions. Flag‐SIRT2, ‐SIRT7, and ‐HDAC2 deacetylases, all found to exist in both the nucleus and cytoplasm, were logically transfected into HEK‐293T cells. The cell lysates were immunoprecipitated for immunoblotting analysis, and the result indicated that SIRT7 could significantly weaken the KHSRP acetylation level (Fig. 2D). Moreover, both Co‐IP and reciprocal Co‐IP assays revealed that KHSRP could interact with SIRT7 (Fig. 2E,F). The endogenous interaction between KHSRP and SIRT7 in LNCaP cells confirmed the same outcome (Fig. 2G). Additionally, silencing SIRT7 could significantly increase endogenous KHSRP acetylation level (Fig. 2H). Therefore, these results indicated that SIRT7 is responsible for the deacetylation of KHSRP.

### 3.3 KHSRP acetylation intrinsically drives tumor growth in PCa

**Fig. 3:**
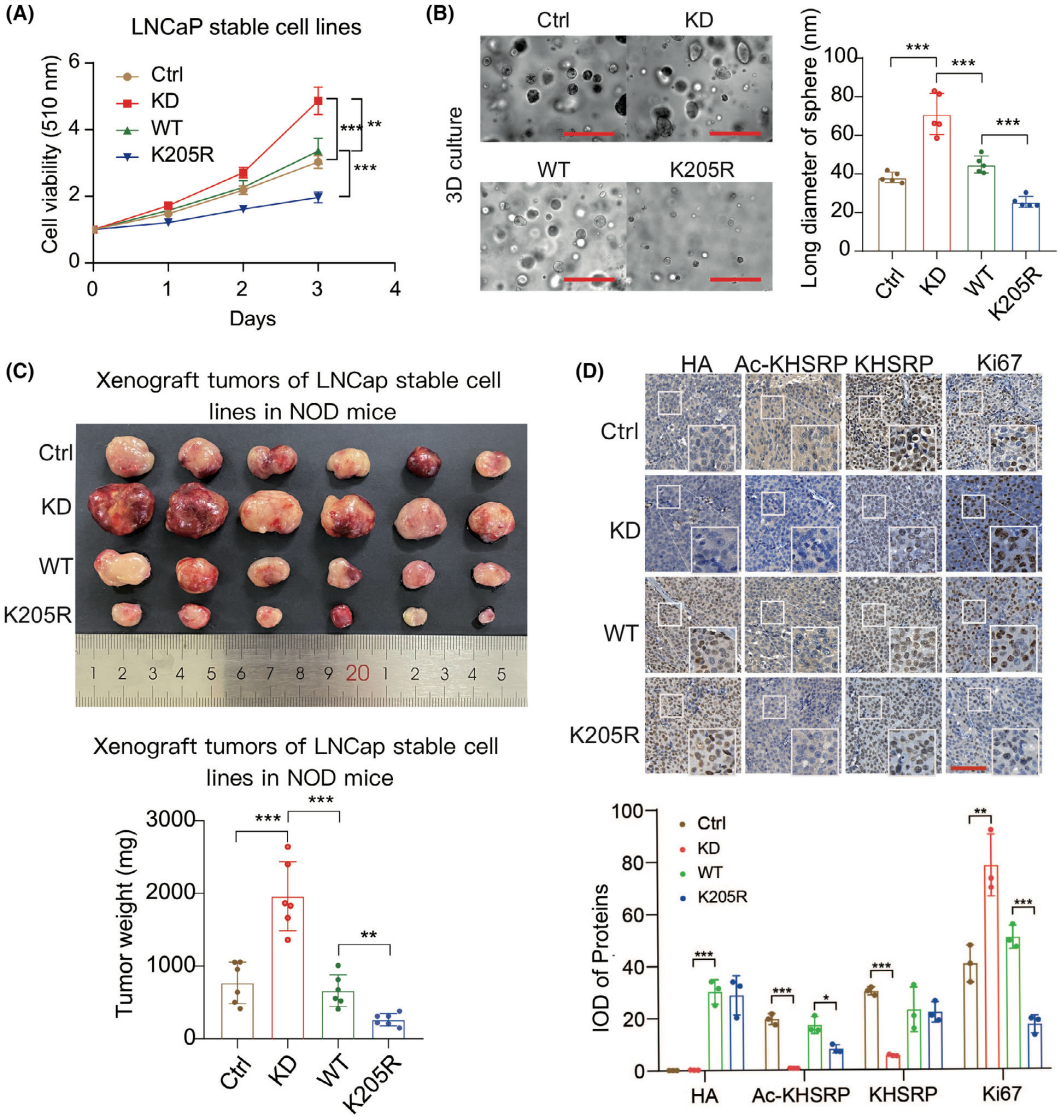
KHSRP acetylation intrinsically drives tumor growth in prostate cancer (PCa). (A) The KHSRP‐silenced and re‐introduced HA‐KHSRP‐WT or HA‐KHSRP‐K205R LNCaP cells were seeded into a 96‐well plate for cell proliferation assay. The empty lentivirus‐infected LNCaP cell was set as the negative control. The growth curve was generated by graphpad prism 8 software. The data are presented as mean ± SD with *t*‐test analysis from three independent experiments, **P < 0.01, ***P < 0.001. (B) The 3D‐culture assay was carried out to detect the growth ability of the indicated LNCaP cell lines. The photos were taken on day 7. Scale bars: 200 μm. The size of 3D cell clumps was measured and statistically columned. The data are presented as mean ± SD with *t*‐test analysis by graphpad prism 8 software from three independent experiments, ***P < 0.001. The scale bars are 200 μm. (C) The stable LNCaP cell lines characterized in (A) were coated with Matrigel and subcutaneously injected into the NOD‐SCID mice. At 4 weeks after injection, tumors from 24 mice were dissected and photographed. The numerical values on the ruler were in centimeters (upper panel). Tumor weight was measured and statistically calculated for the column. The weight of the tumors was presented in columns using graphpad prism 8 software. Data was presented as mean ± SD with *t*‐test analysis, **P < 0.01, ***P < 0.001 (lower panel). (D) Immunohistochemistry staining of xenograft tumors. Xenograft tumors were sliced and stained with antibodies against HA, KHSRP, Ac‐KHSRP, or Ki67. Scale bar = 100 μm (upper panel). Immunohistochemistry results of mouse xenograft tumors were analyzed using image j pro plus software (Bethesda, ML, USA) (lower panel). The staining intensity of three independent determinations is presented as the mean integrated optical density (IOD) values (Mean ± SD). The student *t*‐test was used to analyze the significance between groups (*P < 0.05, **P < 0.01, ***P < 0.001).

To observe the biological function of KHSRP acetylation in PCa, we generated a series of LNCaP stable cell lines using the homemade lentiviral system, as we previously reported [24]. The expression levels of silenced KHSRP, re‐applied HA‐tagged KHSRP‐WT, or KHSRP‐K205R were detected by immunoblotting with anti‐KHSRP or anti‐HA antibody (Fig. S3A). According to the SRB assay, the KHSRP‐silenced cell group exhibited significantly higher proliferation capabilities than the control group. However, re‐applied KHSRP‐WT diminished the growth advantage induced by KHSRP silencing. In contrast, the growth rate of cells harboring KHSRP‐K205R was even lower than that of cells with KHSRP‐WT (Fig. 3A). In 3D culture assay and the xenograft model, KHSRP acetylation also showed a similar biological role *in vivo* and *in vitro* (Fig. 3B,C). Meanwhile, the harvested tumors were subjected to IHC analysis with Ki67 antibody, and the result showed a higher intensity of Ki67 staining in PCa cells with KHSRP‐WT than in those with KHSRP‐K205R (Fig. 3D). In addition, we verified the effectiveness of the homemade KHSRP‐acetyl‐K205 antibody by comparing the staining intense between KHSRP‐silenced tumors and negative control tumors as well as the distinguished KHSRP acetylation level between KHSRP‐WT tumors and KHSRP‐K205R tumors (Fig. 3D). In contrast, the total KHSRP levels did not significantly change in the tumors with HA‐KHSRP‐WT or ‐K205R compared to negative control using the HA or KHSRP antibody (Fig. 3D). These data revealed that acetylated KHSRP in PCa cells mainly contributes to tumor growth.

### 3.4 KHSRP acetylation is mainly related to the PCa tumor progress in the clinical

**Fig. 4:**
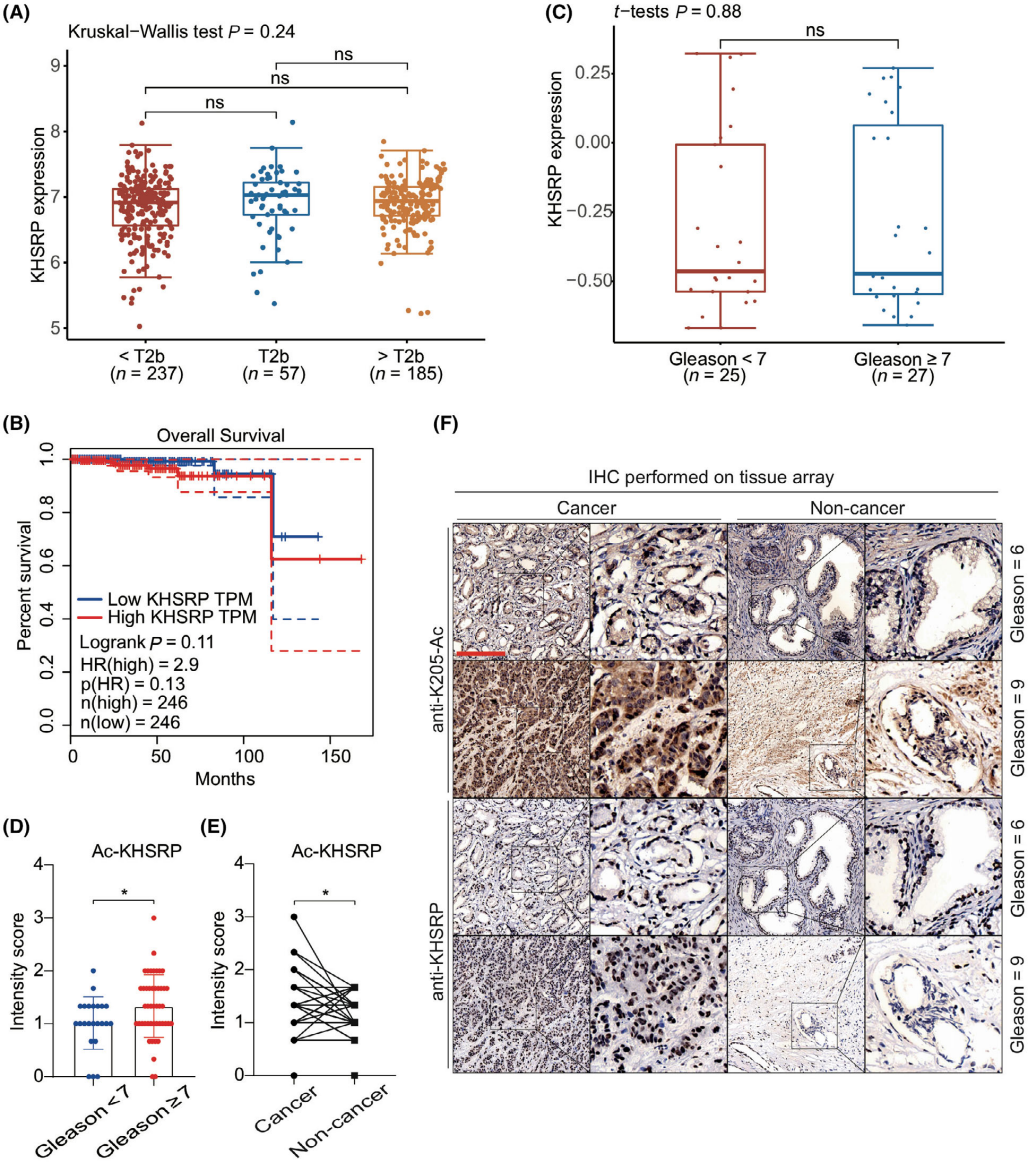
KHSRP acetylation is mainly related to the prostate cancer (PCa) tumor progress in the clinic. (A) A total of 479 PCa samples were adopted from the TCGA database, and KHSRP expression was analyzed among the different clinical tumor stages of PCa using the r package with the Kruskal–Walls test. (B) The overall survival curve was generated by GEPIA web tools (http://gepia.cancer‐pku.cn) containing the TCGA database. The hazard ratio was calculated based on the Cox PH Model. The 95% CI was settled as the dotted line. (C) The KHSRP mRNA transcript levels were compared between PCa patients with low Gleason grade (Gleason score < 7, *n* = 25) and those with high Gleason grade prostate cancer (Gleason score ≥ 7, *n* = 27). The data was from the GEO database (GSE32571). (D–F) Immunohistochemical staining of the prostate tissue array containing 69 pairs of tumor and adjacent tissues was conducted using the indicated antibodies. The intensity scores are presented as columns using graphpad prism 8 software. The data are presented as mean ± SD with *t*‐test analysis, *P < 0.05. The comparison of KHSRP acetylation intensity was conducted between high Gleason score groups (Gleason score ≥ 7, *n* = 23) and low score groups (Gleason score < 7, *n* = 46) (D). The KHSRP‐acetylation intensity between cancer and non‐cancer tissue was compared among 47 pairs of samples with a similar expression of KHSRP (KHSRP score difference between cancer and non‐cancer tissue ≤ 0.33). Data are presented in columns as mean ± SD with paired *t*‐test analysis, *P < 0.05. (E). The representative immunohistochemical images of samples with indicated Gleason score among 69 selected pairs of PCa tissues were presented in columns 1 and 3. Magnified areas were presented in columns 2 and 4. Scale bar: 200 μm (F).

More intriguingly, the KHSRP mRNA expression was related to neither the clinical tumor stages of PCa nor the patient's overall survival (Fig. 4A,B). In addition, there was no difference in the expression of KHSRP between patients with low and high Gleason grade PCa (Fig. 4C). Since KHSRP acetylation significantly affects the tumor growth ability of prostate cancer cells, we tried to verify whether KHSRP acetylation is variated in PCa tissue samples. The immunohistochemistry analysis with KHSRP antibody or homemade KHSRP‐acetyl‐K205 antibody revealed that KHSRP acetylation in tissues with Gleason score ≥ 7 was higher than that in tissues with Gleason score < 7 (Fig. 4D), suggesting that acetylated KHSRP is related to the malignancy of PCa. To further exclude the variation of KHSRP acetylation level caused by the overall KHSRP expression, we screened the samples with a similar term of KHSRP (The difference of KHSRP intensity score between cancer and non‐cancer samples ≤ 1/3). The intensity score of KHSRP acetylation in these samples showed that KHSRP acetylation in cancer tissues is higher than that in non‐cancer tissues, indicating that KHSRP acetylation may promote the tumorigenesis of PCa (Fig. 4E,F). These results suggested that KHSRP acetylation might be critical in driving PCa growth and malignancy.

### 3.5 KHSRP acetylation tightly responds to androgen stimuli or AR activity in PCa

**Fig. 5:**
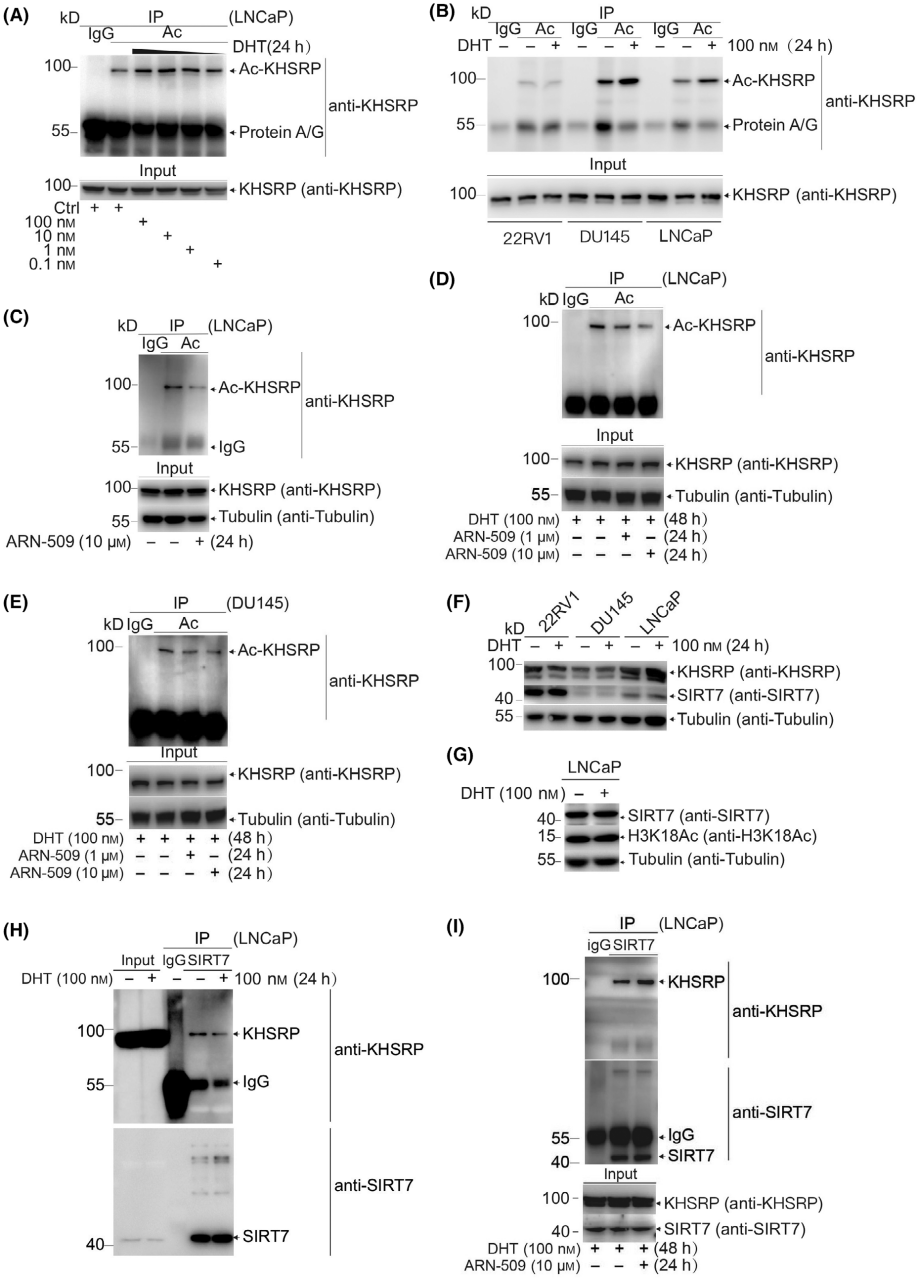
KHSRP acetylation tightly responds to androgen stimuli or androgen receptor (AR) activity in PCa. (A) LNCaP cells were treated with dihydrotestosterone (DHT) with indicated concentrations for 24 h, and then cell lysates were immunoprecipitated for the KHSRP acetylation assay. (B) 22RV1, DU145, and LNCaP cells were lysed after treatment with 100 nm DHT for 24 h, and cell lysates were immunoprecipitated for KHSRP acetylation detection. (C) LNCaP cells were treated with the AR inhibitor ARN‐509 (10 μm) for 24 h, and cell lysates were immunoprecipitated for KHSRP acetylation detection. (D, E) LNCaP or DU145 cells stimulated with 100 nm DHT for 24 h were treated with additional ARN‐509 for another 24 h. Cell lysates were subject to SDS/PAGE gel resolving and western blotting analysis after immunoprecipitation assay. (F) After 24 h of treatment with DHT (100 nm), SIRT7 expression was determined in lysates from 22RV1, LNCaP, or DU145 cells by western blotting. (G) After 24 h of treatment with DHT (100 nm), SIRT7 and H3K18Ac expression was determined in lysates LNCaP cells by western blotting. (H, I) LNCaP cells were treated with DMSO or 100 nm DHT for 24 h (H), or LNCaP cells treated with DHT were treated with additional ARN‐509 (I). After that treatment, cell lysates were immunoprecipitated with anti‐SIRT7 or normal rabbit IgG. The immunoprecipitates were resolved in SDS/PAGE and detected with indicated antibodies by western blotting. The representative result of three independent experimentals was displayed.

Considering that AR plays a crucial role in PCa development and progression, we intended to identify whether the pro‐oncogenic effect of KHSRP acetylation is related to the AR signaling pathway. By treating androgen‐dependent LNCaP cells with different concentrations of dihydrotestosterone (DHT), an active form of androgen, we found that KHSRP acetylation could be significantly induced in a dose‐dependent manner (Fig. 5A). Importantly, 1 nm DHT was the median DHT level in the blood of PCa patients, and a high DHT concentration is related to worse PCa malignancy [25]. KHSRP acetylation could be induced upon androgen stimuli in the androgen‐dependent or independent cell lines LNCaP or DU145 but not 22RV1 cells (Fig. 5B), suggesting that the regulatory function of KHSRP acetylation in response to androgen stimuli is complex in different types of PCa cells. One possible reason is the sensitivity difference among variant or non‐variant AR upon androgen stimuli in those diverse cell lines [26]. Intriguingly, KHSRP acetylation was significantly suppressed in LNCaP cells treated with ARN‐509, the androgen receptor inhibitor (Fig. 5C). Moreover, the KHSRP acetylation induced by DHT can be reduced again under the additional ARN‐509 treatment in both LNCaP and DU145 cells (Fig. 5D,E), confirming that DHT‐induced alteration of KHSRP acetylation is tightly associated with AR regulation. It was worth noting that the higher KHSRP acetylation level was accompanied by the lower SIRT7 expression (Fig. 5B,F). To address how DHT affects KHSRP acetylation, we verified that neither the enzymatic activity nor the protein level of SIRT7 in LNCaP cells was impaired after DHT treatment on account of the unchanged acetylation level of histone protein H3K18, a specific substrate of SIRT7 [27] (Fig. 5F,G). However, the interaction between KHSRP and SIRT7 was attenuated upon the DHT stimulation (Fig. 5H and Fig. S3B) and recovered by additional ARN‐509 treatment (Fig. 5I). These results revealed that KHSRP acetylation is closely related to androgen stimulation and AR regulation by impairing the binding ability of SIRT7 to KHSRP.

### 3.6 KHSRP acetylation is highly associated with DNA damage response

**Fig. 6:**
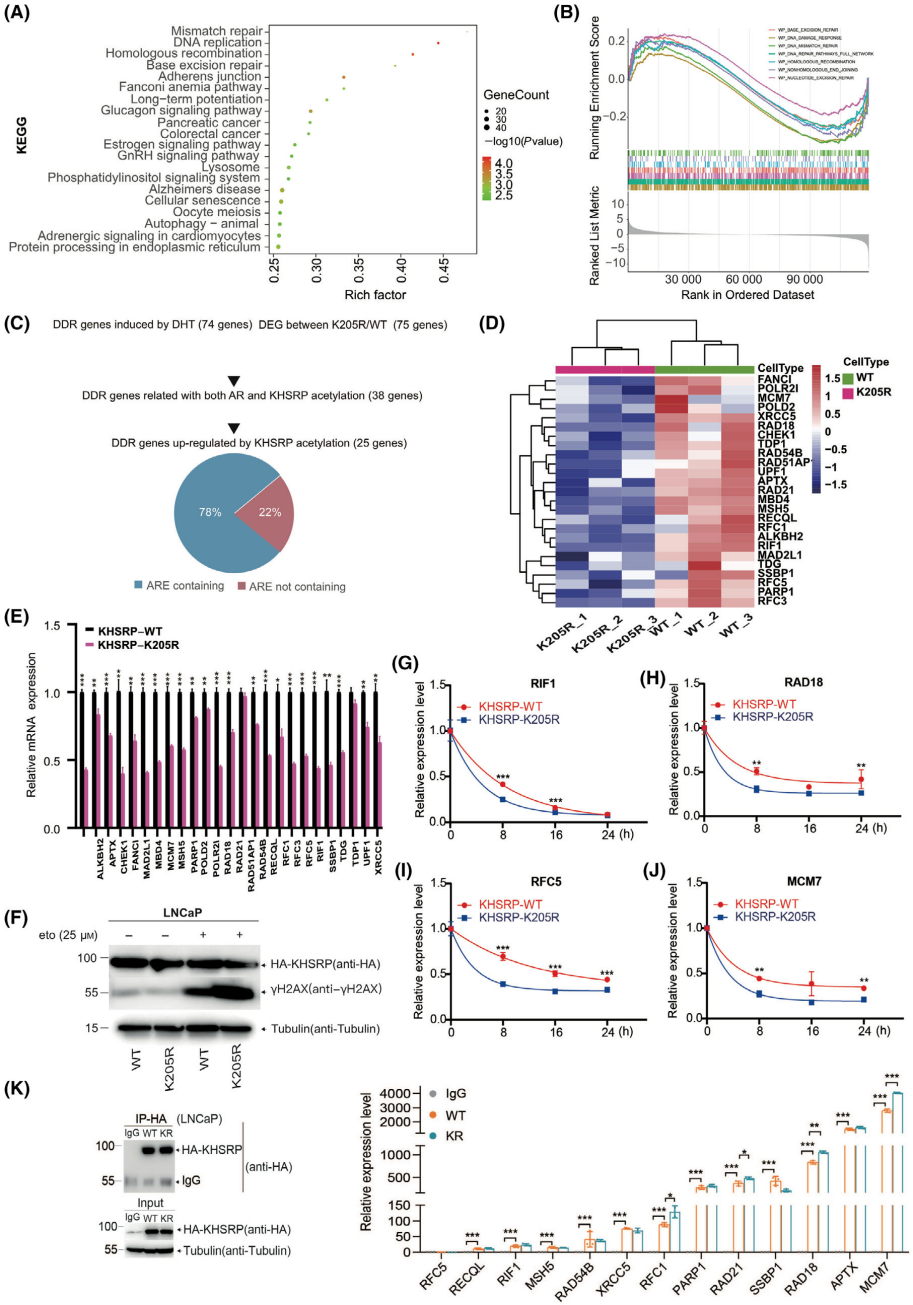
KHSRP acetylation is highly associated with DNA damage response (DDR). (A, B) Differentially expressed mRNAs between LNCaP stable cell lines harboring KHSRP‐WT or ‐K205R were enriched and analyzed based on a KEGG pathway analysis (A). The relationship between the differentially expressed mRNAs and DDR pathways was analyzed by GSEA analysis (B). (C) A total of 38 common genes were screened from the DDR genes induced by both androgen and KHSRP acetylation. Twenty‐five upregulated genes by KHSRP acetylation were further screened out. (D) The screened 25 DDR genes induced by both androgen and KHSRP acetylation were generated with the heatmap. (E) The mRNA expression of the 25 selected DDR genes was assessed using the method of qRT‐PCR. The data are presented as mean ± SD with *t*‐test analysis from three independent experiments, *P < 0.01, **P < 0.01, ***P < 0.001. (F) γ‐H2AX expression was determined in LNCaP cell lines harboring HA‐KHSRP‐WT and K205R upon treatment with etoposide (25 μm) for 24 h by immunoblotting assay with indicated antibodies. The result was representative of three independent replicates. (G–J) LNCaP cells were treated with Actinomycin D (5 μg·mL^−1^) for 2 h before time 0. Subsequently, LNCaP‐KHSRP‐WT/KR cells treated with Actinomycin D for an additional 0, 8, 16, and 24 h were collected for the qRT‐PCR analysis. The expression level of each gene at each time point was presented as a remaining percentage compared with time 0. Data was plotted using graphpad prism by one‐phase decay mode of non‐linear regression (curve fitting). The data are presented as mean ± SD with *t*‐test analysis from three independent experiments, **P < 0.01, ***P < 0.001. (K) LNCaP stable cell lines were lysed with lysis buffer (150 mm KCl, 25 mm pH 7.4 Tris–HCl, 5 mm pH 8.0 EDTA, 0.5% NP‐40, 0.5 mm DTT, 100 U·mL^−1^ RNase inhibitor, and 1% protease inhibitor) and incubated with HA antibody‐conjugated magnetic beads overnight at 4 °C. After five times washing, one‐tenth of the beads were used for western blot to detect KHSRP protein pull‐down efficiency (left panel), and the rest of the beads were used for the qRT‐PCR detection of DDR gene expression associated with HA‐KHSRP protein binding (right panel). The data are presented as mean ± SD, and the statistical analysis was conducted using *t*‐test across three independent experiments, *P < 0.05, **P < 0.01, ***P < 0.001.

RNA‐seq was conducted in the stable LNCaP cell lines to determine the underlying mechanism of KHSRP acetylation in PCa tumorigenesis. Data analysis showed no valuable difference in miRNA biogenesis in cell lines between KHSRP‐WT and KHSRP‐K205R (Fig. S4). However, KEGG enrichment analysis of differentially expressed mRNA transcripts suggested that KHSRP acetylation‐associated genes were significantly enriched in clusters of Homologous recombination, Mismatch repair, Base excision repair, or Fanconi anemia (Fig. 6A). Intriguingly, all those clusters are the crucial components of the DNA damage response (DDR) pathway. Furtherly, we analyzed the differential gene datasets by GSEA and found that these genes were significantly enriched in the DNA repair and damage response‐related pathways (Fig. 6B). Since AR has been reported to induce expression of DNA repair genes [28], and KHSRP acetylation is coincidentally related to AR response, we naturally hypothesize that AR‐regulated DNA damage response may account for KHSRP acetylation. By aligning differentially expressed genes regulated by KHSRP acetylation with androgen‐induced DDR genes from the previous report [28], we surprisingly found a common list of 38 genes (Table S2).

Moreover, a total of 25 downregulated genes due to the deacetylation in cells were further screened and validated by qRT‐PCR (Fig. 6C–E). Intriguingly, 78% of these 25 genes contain ARE elements in the 3′‐UTR of their mRNAs (Fig. 6C and Table 1). To verify further, we found that the LNCaP cells with KHSRP‐K205R presented a higher expression of the specific DNA damage marker γ‐H2AX than those with KHSRP‐WT upon etoposide treatment (Fig. 6F). The qRT‐PCR analysis proved the most mRNA of DDR genes degraded faster in LNCaP‐K205R cells than that in LNCaP‐WT cells (Fig. 6G–J and Fig. S5A–L). To trace the binding ability of KHSRP to mRNAs, the RIP assay revealed that KHSRP significantly binds to the target mRNAs by qRT‐PCR detection after immunoprecipitation, particularly the KHSRP‐K205R even which promotes the mRNA decay in PCa cells (Fig. 6K). These demonstrated that KHSRP acetylation functions as a downstream regulator of AR and is highly associated with DNA damage response through impeding KHSRP‐regulated mRNA decay.

**Table 1:** The mRNA level of DDR genes and ARE consensus analysis.

Gene symbol	log2Fold change	*P*‐value	ARE	
			Num.	Consensus (red label)
ALKBH2	−1.034692048	0.0001392860	Null	/
APTX	−0.784623032	0.0344756510	2	CACAA **ATTTA** TTCTA
CHEK1	−0.687565841	0.0057937680	1	TTCCA **ATTTA** TTTTG
FANCI	−1.561488453	0.0002959490	2	TTCTG **ATTTA** CTTGT
MAD2L1	−0.381103256	0.0349528570	11	CATGA **ATTTA** TTGCA
MBD4	−2.105604939	0.0000000003	1	TATAT **ATTTA** AAAAA
MCM7	−2.771812223	0.0200371610	Null	/
MSH5	−2.113645974	0.0000001980	Null	/
PARP1	−0.25392718	0.0248666080	1	ATACT **ATTTA** GATTT
POLD2	−0.825922184	0.0420094630	Null	/
POLR2I	−0.310788894	0.0488854300	Null	/
RAD18	−2.433783266	0.0144478800	8	AAGAA **ATTTA** TGATT
RAD21	−0.20923922	0.0000111000	4	GTTTA **ATTTA** AAACT
RAD51AP1	−3.245648393	0.0467419350	4	TTTAT **ATTTA** CATTG
RAD54B	−1.693269106	0.0004121380	5	AAGCT **ATTTA** TGGCA
RECQL	−0.242384411	0.0198644550	2	AACAT **ATTTA** TGTTT
RFC1	−1.08034092	0.0463407140	5	CTGGA **ATTTA** GATGT
RFC3	−0.352778699	0.0000539000	7	AGGAG **ATTTA** CACAT
RFC5	−0.888966593	0.0069535500	7	CAGGC **ATTTA** AAAAG
RIF1	−10.10402023	0.0024179140	23	GCAGA **ATTTA** CTAAG
SSBP1	−1.278927643	0.0354741280	Null	/
TDG	−1.93971224	0.0465848560	Null	/
TDP1	−0.378481333	0.0335728650	3	GTGAA **ATTTA** AGTGT
UPF1	−0.239964813	0.0470835640	3	CCTTC **ATTTA** AAGAA
XRCC5	−0.458643659	0.0003235120	2	GAGAA **ATTTA** CTACA

## 4 Discussion

Cancer cells have the characteristic of genomic instability and are usually accompanied by defects in the DDR pathway, making it easier to accumulate more damaged DNA than normal cells [29, 30]. Defects in the DDR pathway have been a prominent feature, with about 10% of primary tumors and 25% of distant metastases in PCa [31, 32]. Clinical practice shows that the development and progression of PCa can be driven by AR activity at all stages of PCa. The most practical treatment option for locally advanced and metastatic PCa is ADT or direct AR antagonism [33, 34]. Although these treatments are initially effective, resistant tumors eventually emerge through multiple mechanisms with no durable therapeutic options [33]. Increasing evidence has revealed that PCa events are closely related to AR‐regulated DDR, and targeting DDR disorder shows an attractive prospect for clinical therapy [30, 33, 34, 35, 36]. However, the biological relevance or precise mechanism between DDR and AR activity is still poorly understood.

This work reports that KHSRP, a multifunctional single‐strand nucleic acid‐binding protein, can be significantly acetylated *in vitro* and *in vivo* (Fig. 1), and SIRT7 is a direct deacetylase of KHSRP (Fig. 2). As reported literature, KHSRP is required for miRNA biogenesis and mRNA decay in cells [15, 16, 17]. KHSRP deficiency can cause multiple physiological abnormalities and even cancer disease [17]. However, we find that the aberrant acetylation of KHSRP is the authentic and intrinsic driver for tumor growth in PCa (Fig. 3A–C), which is also confirmed in PCa patient samples according to the Gleason Score (Fig. 4D,E). Those data reveal that KHSRP acetylation may play a critical role in the development and progression of PCa. More importantly, KHSRP acetylation can either be induced upon the androgen stimuli or depressed by the AR inhibitor (Fig. 5). A possible reason is the alternated binding ability to KHSRP of the deacetylase SIRT7 alone with the AR activity change (Fig. 5H,I). To some extent, these shreds of evidence explain the scientific nature and effectiveness of the androgen deprivation therapy for PCa in clinical. Moreover, the acetylation level of KHSRP in androgen‐independent DU145 cells was significantly higher than that in androgen‐dependent LNCaP cells (Fig. 5D), which seems to indicate that KHSRP acetylation is a necessary factor in PCa occurrence and development and AR activity may function as a regulating switch in this process.

More intriguingly, androgen can stimulate KHSRP acetylation in androgen‐dependent LNCaP cells and even more in androgen‐independent DU145 cells (Fig. 5A–E). It is traditionally considered that DU145 cells do not contain the expression of AR; however, Alimirah et al. [26] reported that DU145 and PC3 cells do not express full‐length AR but express the truncated AR, which could not be recognized by the conventional antibodies. According to our present data, therefore, the truncated AR in DU145 cells may also respond to the androgen stimuli, but the response pattern may differ from that of the full‐length AR in LNCaP cells. Oddly, KHSRP acetylation in another androgen‐dependent 22RV1 cell with full‐length AR still cannot be induced by androgen stimulation (Fig. 5B). The great possibility is that SIRT7 expression is much higher in 22RV1 than in LNCaP or DU145 cells (Fig. 5F), resulting to androgen cannot efficiently induce the KHSRP acetylation. All these hints that SIRT7 may have a tremendous biological link within both androgen‐dependent and ‐independent PCa cells, at least, the SIRT7‐KHSRP‐DDR signaling.

AR signaling in PCa cells has been connected with numerous aspects of DDR pathways, including ATM‐Chk2 regulated signaling for DDR initiation [37], poly(ADP‐ribose) polymerase function [38], and non‐homologous end‐joining recombination [28]. Alterations in DDR pathways are thought to be associated with the risk of PCa development, progression, and aggressiveness [30]. DDR defects have been applied for the common treatment in patients with advanced ovarian cancer, like Platinum‐based therapies combined with Paclitaxel, which is aimed to cause DNA inter‐ and intra‐strand crosslinks, and the defective DDR system cannot repair such damage [39]. The inhibitor of PARP, an essential gene in the DDR pathway, has also been approved to treat BRCA1/2 deficient PCa [12]. In this present study, we revealed that KHSRP acetylation involves a significant DDR response and could specifically down‐regulate the expression of DDR‐relative genes (e.g., PARP1, CHEK1, FANCI, RAD21, and RAD54B) (Fig. 6D). Since KHSRP acetylation can respond to androgen stimuli or AR activity (Fig. 5), we believe that KHSRP acetylation may play a critical regulatory role in the AR‐mediated DDR and is required for tumor growth in PCa.

KHSRP is an essential regulator of precursor miRNA processing, but we did not observe the miRNA alteration resulting from KHSRP acetylation (Fig. S4). Alternatively, KHSRP acetylation mainly impairs its mRNA decay function and tends to regulate the expression of specific genes by impeding the bind with the AREs in the 3′‐UTR regions of the DDR‐related genes (Fig. 6C and Table 1), which is because the deacetylation on KHSRP can enhance its affinity to the specific mRNAs or the ability to recognize and decay mRNA substrates (Fig. 6K).

## 5 Conclusions

**Fig. 7:**
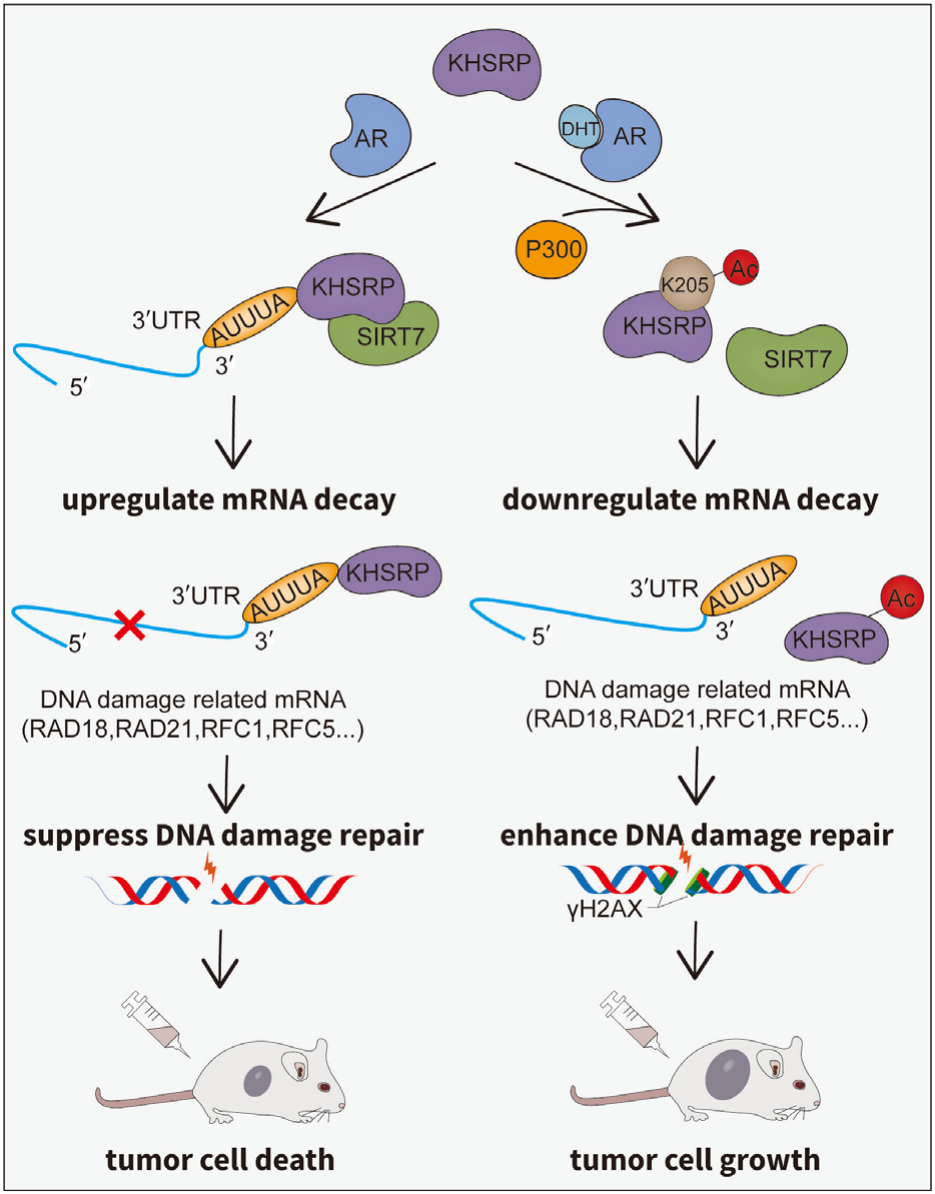
A model depicting the regulatory role of KHSRP acetylation in prostate cancer (PCa). In general, KHSRP, under the regulation of deacetylase SIRT7, maintains a relatively low acetylation level and feasibly decays the mRNAs of DNA damage response (DDR) genes to weaken the DNA repair ability, resulting in the death of PCa cells. The interaction between KHSRP and SIRT7 is attenuated due to some disorder factors such as androgen receptor (AR) activation in PCa, which leads to acetylated KHSRP being disabled to efficiently attach the adenine uridine (AU)‐rich elements (AREs) in the 3′‐UTR region of DDR genes to exert mRNA decay function and finally promotes the tumorigenesis of PCa cells. This study reveals a new mechanism of AR‐mediated DDR in PCa and provides novel insight into KHSRP biology and a potential therapeutic strategy for castration‐resistant PCa.

Our study found that KHSRP acetylation as a downstream regulator of AR can widely promote the expression of DDR‐related genes, especially the AREs‐containing genes, to drive tumor growth in PCa (Fig. 7). This study sheds light on a novel insight into KHSRP biology to deeply comprehend the mechanism of PCa development, and it provides an attractive strategy for PCa treatment, particularly castration‐resistant PCa, targeting KHSRP acetylation in the AR‐mediated DDR pathway.

## Conflict of interest

The authors declare no conflict of interest.

## Author contributions

HY and RC performed most of the experiments and wrote the manuscript; BC and QW helped with all experiments and contributed to the implementation and interpretation of the results; MW, JA, and WA analyzed the RNA‐seq data; YT helped with the Animal Xenograft experiment; JY gave the academic suggestion and technical guidance. MX and HY designed and guided the whole study; MX, BJ, YZ, and HY contributed to the project implementation, discussion of the results, providing the ethics committee approval, and responding the peer‐review comment; MX is responsible for manuscript revision, editing, and final draft; All authors read and approved the final manuscript.

### Peer review

The peer review history for this article is available at https://www.webofscience.com/api/gateway/wos/peer‐review/10.1002/1878‐0261.13634.

## Supporting information


**Fig. S1.** Nine sites of KHSRP acetylation were identified by mass spectrometry.

**Fig. S2.** Identification of the specificity of the homemade anti‐KHSRP‐K205‐Ac antibody.

**Fig. S3.** Identification LNCaP stable cell lines.

**Fig. S4.** KHSRP acetylation has no significant impact on miRNA biogenesis.

**Fig. S5.** The mRNA decay assay on DNA damage response‐related genes.

**Table S1.** All primers or oligonucleotides were used in this study.
**Table S2.** Common gene list between KHSRP acetylation‐regulated genes and dihydrotestosterone (DHT)‐induced genes in LNCaP cells.

## Data Availability

All data or datasets used and analyzed in this study are available on reasonable requirements from the corresponding authors.
