# Supplementary figures and images for: Acetylated KHSRP impairs DNA‐damage‐response‐related mRNA decay and facilitates prostate cancer tumorigenesis
Source: Mol Oncol. 2024 Mar 19;18(9):2314–30. doi: 10.1002/1878-0261.13634 (PMC11467790; doi:10.1002/1878-0261.13634)

# Figure S1

K87

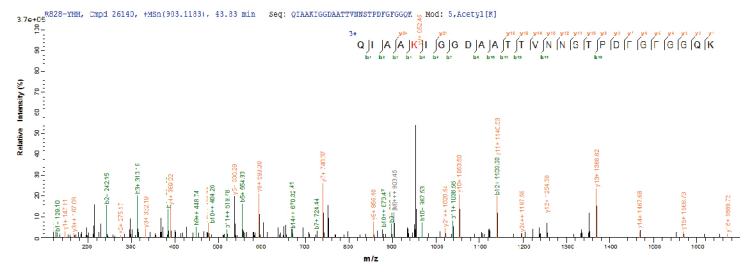

K266

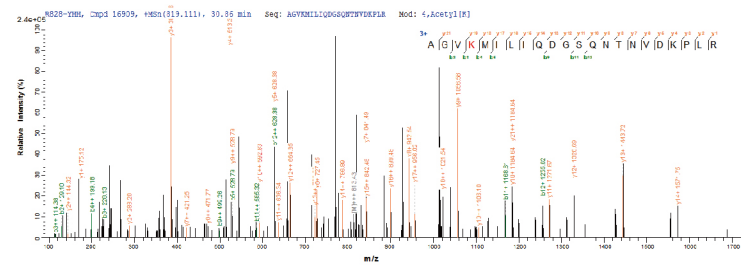

K109

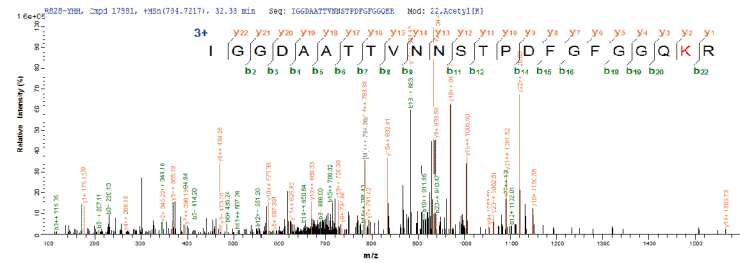

K291

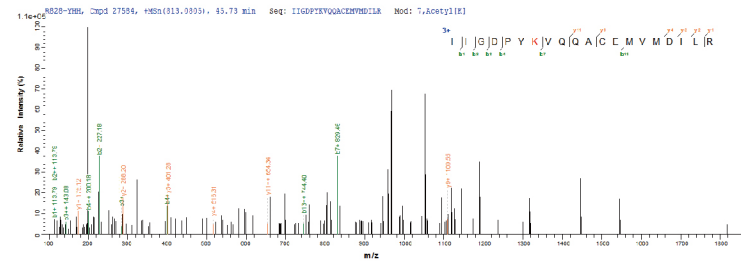

K177

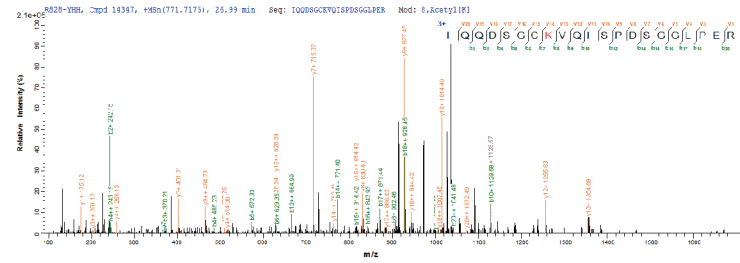

K359

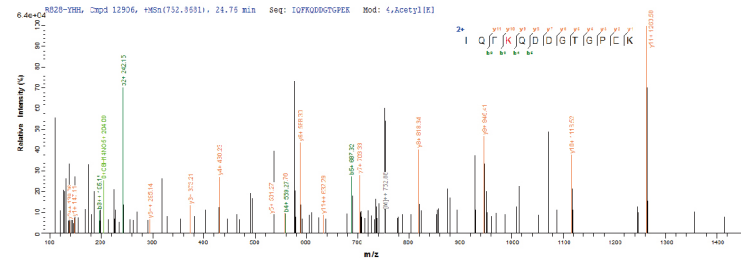

K205

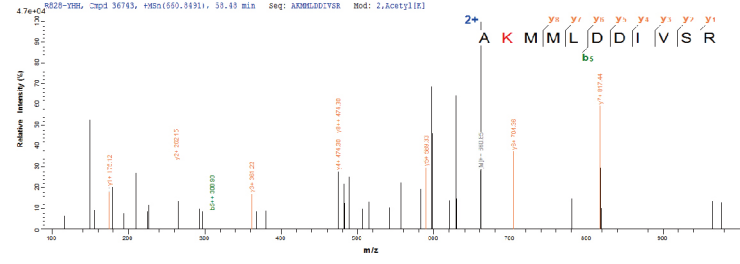

K654

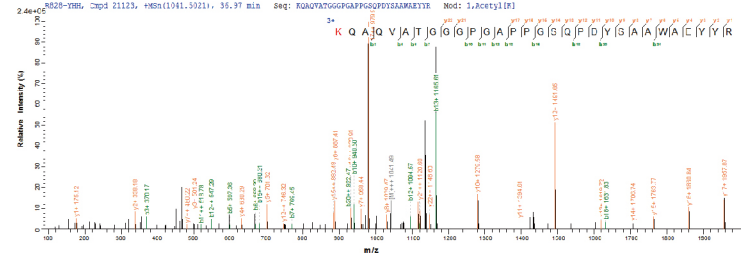

K251

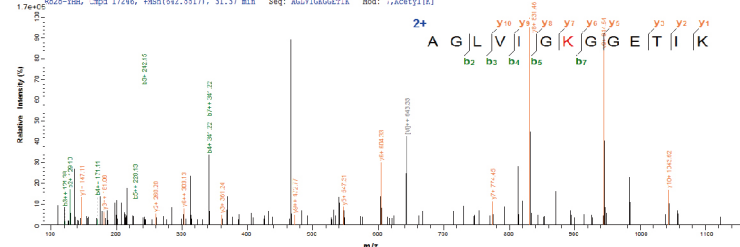

Supplement: Supplementary file 1 — Fig. S1. Nine sites of KHSRP acetylation were identified by mass spectrometry. [file MOL2-18-2314-s004.pdf]

# Figure S2

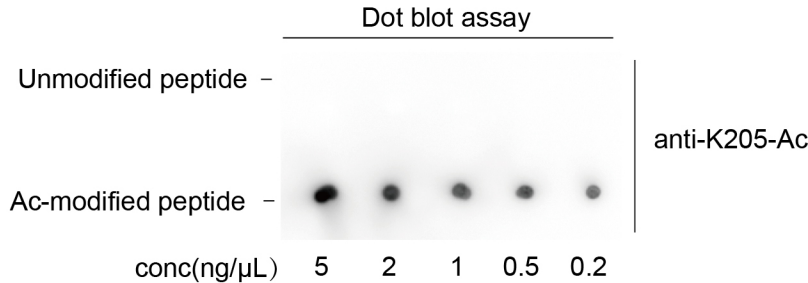

Supplement: Supplementary file 2 — Fig. S2. Identification of the specificity of the homemade anti‐KHSRP‐K205‐Ac antibody. [file MOL2-18-2314-s005.pdf]

# Figure S3

**a**

LNCaP

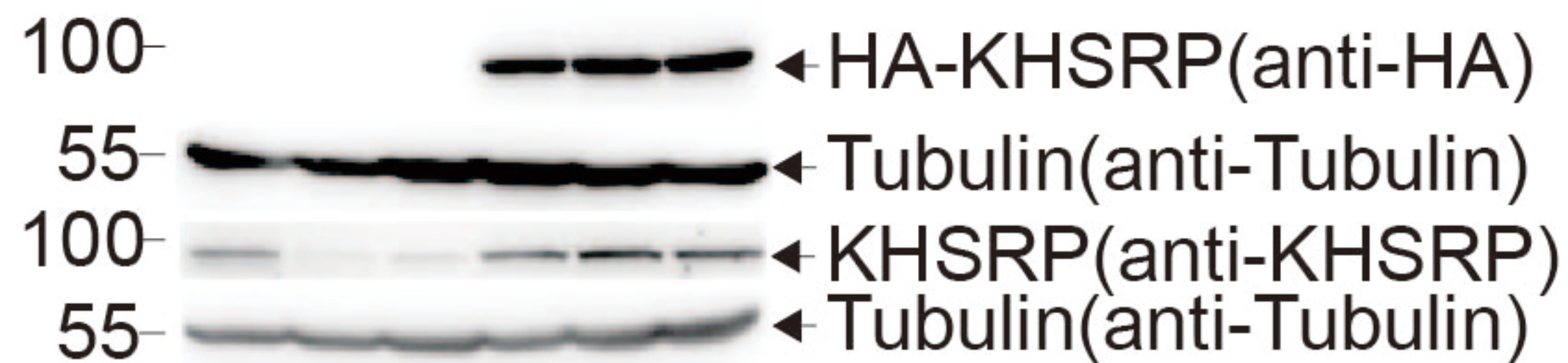

Ctrl  
sh-KHSRP-UTR  
sh-KHSRP-CDS  
HA-KHSRP-WT  
HA-KHSRP-K205R  
HA-KHSRP-K205Q

**b**

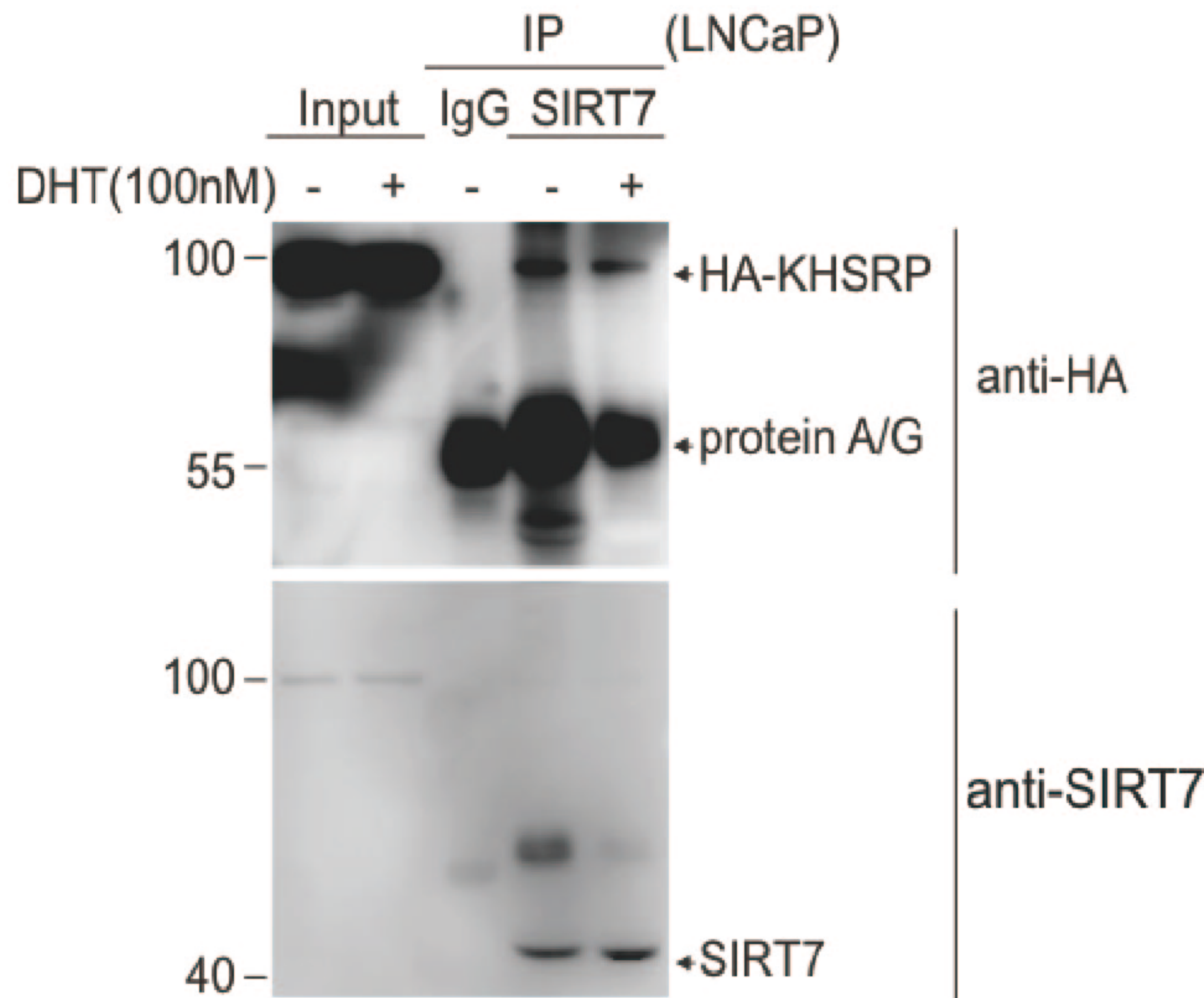

Supplement: Supplementary file 3 — Fig. S3. Identification LNCaP stable cell lines. [file MOL2-18-2314-s002.pdf]

Figure S4

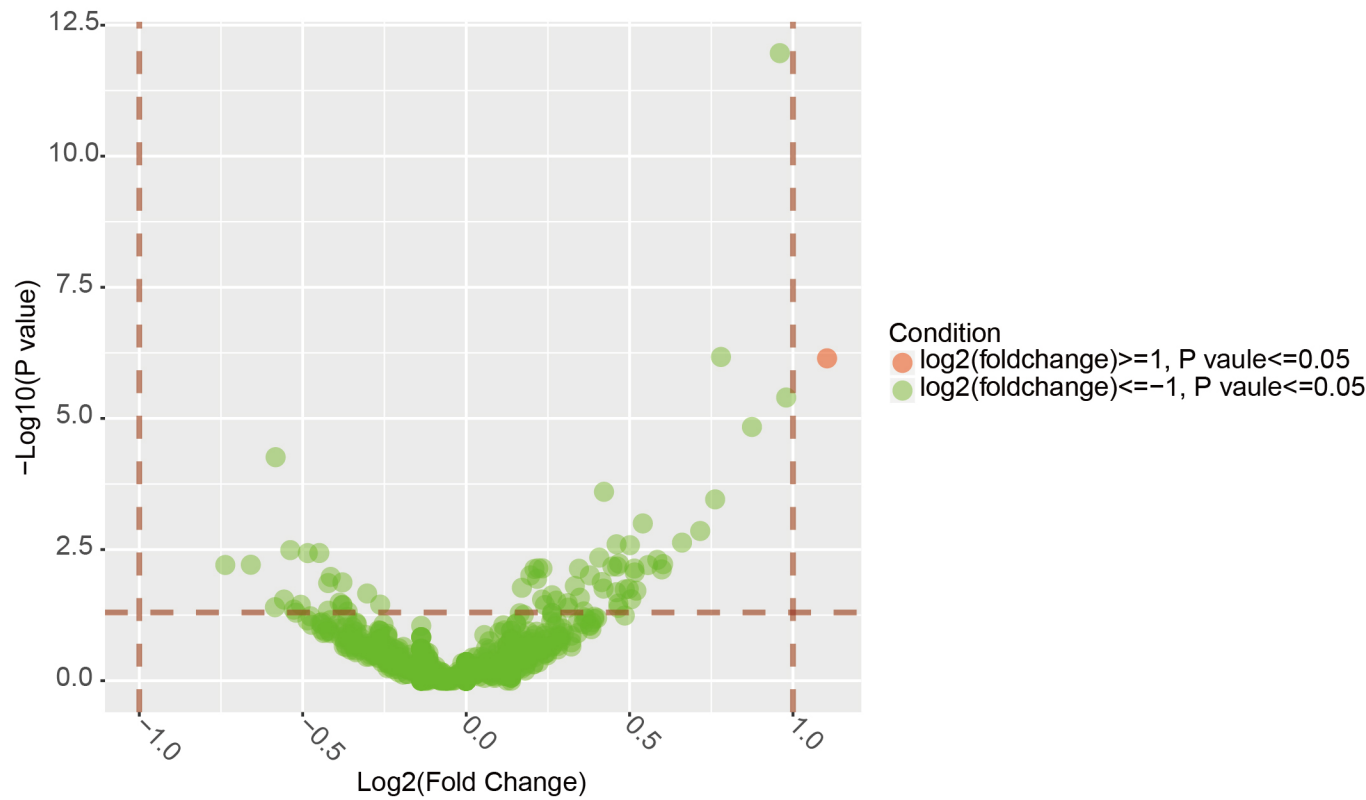

Supplement: Supplementary file 4 — Fig. S4. KHSRP acetylation has no significant impact on miRNA biogenesis. [file MOL2-18-2314-s006.pdf]

Figure S5

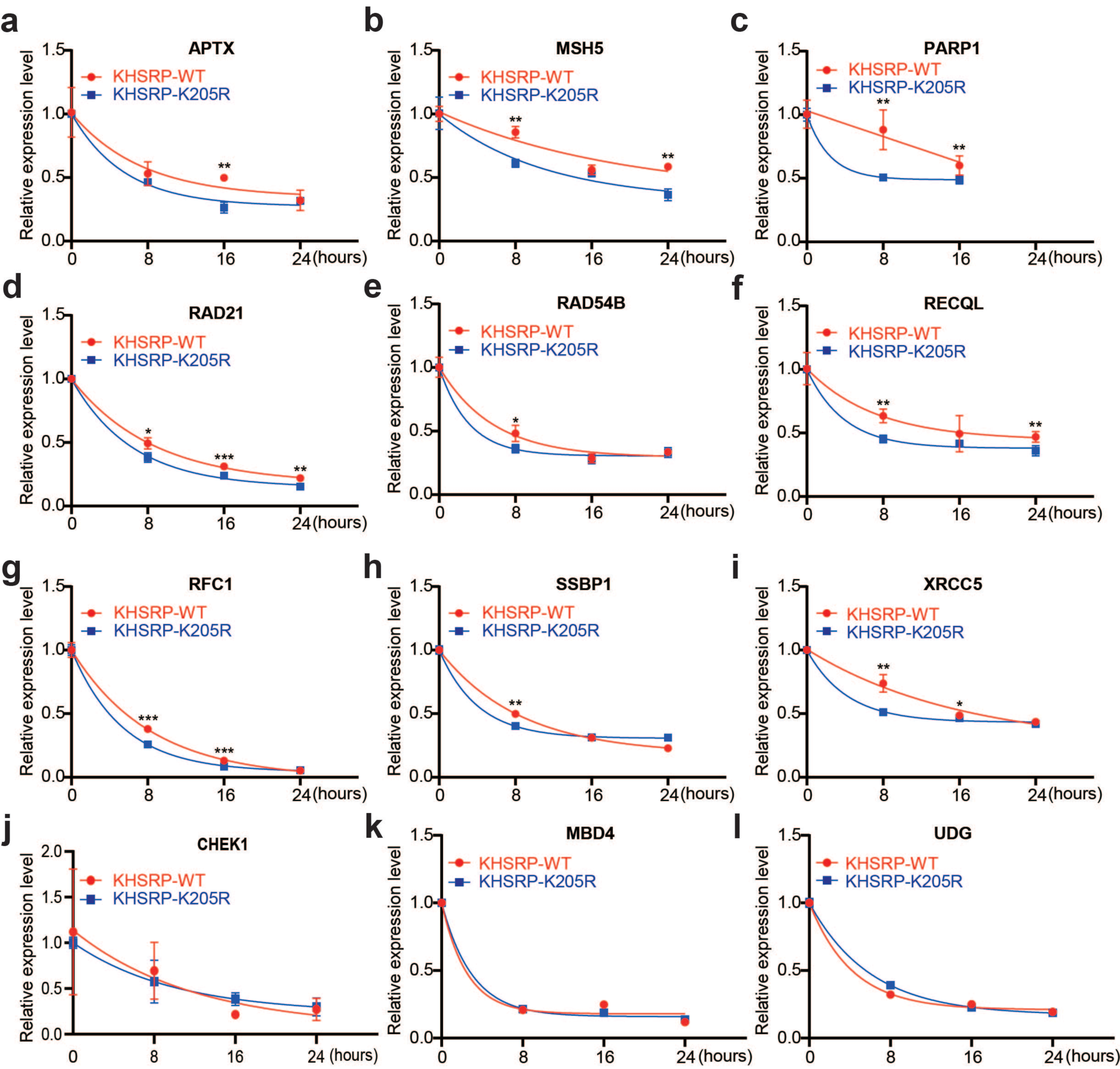

Supplement: Supplementary file 5 — Fig. S5. The mRNA decay assay on DNA damage response‐related genes. [file MOL2-18-2314-s001.pdf]
