# Supplementary material for: Acetylated KHSRP impairs DNA‐damage‐response‐related mRNA decay and facilitates prostate cancer tumorigenesis
Source: Mol Oncol. 2024 Mar 19;18(9):2314–30. doi: 10.1002/1878-0261.13634 (PMC11467790; doi:10.1002/1878-0261.13634)
Supplement: Supplementary file 6 — Table S1. All primers or oligonucleotides were used in this study. Table S2. Common gene list between KHSRP acetylation‐regulated genes and dihydrotestosterone (DHT)‐induced genes in LNCaP cells. [file MOL2-18-2314-s003.docx]

**Table S1. All primers or oligonucleotides were used in this study.**

|  | genes | primers | sequence (5’-3’) |
| --- | --- | --- | --- |
| **Primers used for the construction of HA-P300 and Flag-KHSRP** | HA-P300 | F | gttccagactatgcaggatccatggccgagaatgtggtgga |
|  |  | R | tgctggatatctgcagaattcctagtgtatgtctagtgtac |
|  | Flag-KHSRP | F | cggaattcatgtcggactacagcacggg aggac |
|  |  | R | ccgctcgagtcattgagcctgctgctgtccctgc |
| **Primers used for constructions of lentiviral KHSRP expression, shRNAs for KHSRP-CDS, -UTR, and shRNA for SIRT7** | KHSRP-shUTR | F | gatccgctgaggataaagcaattcattctcgagaatgaattgctttatcctcagctttttg |
|  |  | R | aattcaaaaagctgaggataaagcaattcattctcgagaatgaattgctttatcctcagcg |
|  | KHSRP-shCDS | F | gatccgcccgagaagattgctcatatactcgagtatatgagcaatcttctcgggctttttg |
|  |  | R | aattcaaaaagcccgagaagattgctcatatactcgagtatatgagcaatcttctcgggcg |
|  | Re-applied HA-KHSRP | F | ttttgacctccatagaagattatgggtacctatccttacga |
|  |  | R | gcgtctaggaagagctcgatcagttagaggagaacttgtct |
|  | SIRT7-shUTR | F | gatccgagaacggaactcgggttatttctcgagaaataacccgagttccgttctctttttg |
|  |  | R | aattcaaaaagagaacggaactcgggttatttctcgagaaataacccgagttccgttctcg |
| **qRT-PCR primers for DDR-relative genes** | ALKBH2 | F | gggagcaccgagatgatgaaa |
|  |  | R | ccttatgccggaagacaaagt |
|  | APTX | F | caaaggcccgttaccattgg |
|  |  | R | acccagcaaaatctacaatcacc |
|  | CHEK1 | F | atatgaagcgtgccgtagact |
|  |  | R | tgcctatgtctggctctattctg |
|  | FANCI | F | ccacctttggtctatcagcttc |
|  |  | R | caacatccaatagctcgtcacc |
|  | GAPDH | F | ggagcgagatccctccaaaat |
|  |  | R | ggctgttgtcatacttctcatgg |
|  | MAD2L1 | F | gttcttctcattcggcatcaaca |
|  |  | R | gagtccgtatttctgcactcg |
|  | MBD4 | F | ccgtcacctctagtgagcg |
|  |  | R | gcagaagcgatgggttcttgta |
|  | MCM7 | F | gcctgtgggaaatatccctcg |
|  |  | R | gtaccacctgtcggaaccc |
|  | MSH5 | F | agtgactccactatccacttcat |
|  |  | R | actggggattgatctcatcca |
|  | PARP1 | F | cggagtcttcggataagctct |
|  |  | R | tttccatcaaacatgggcgac |
|  | POLD2 | F | ccacccgcctcatccaaat |
|  |  | R | ccaagaccagctcgtcatctg |
|  | POLR2I | F | ggcttcgtgggtattcgctt |
|  |  | R | ctcctgctggtaatcacagttc |
|  | RAD18 | F | gagccggatctgaaaaataaccg |
|  |  | R | gctggtgactctaaagcaaactg |
|  | RAD21 | F | ggataagaagctaaccaaagccc |
|  |  | R | ctcccagtaagagatgtcctgat |
|  | RAD51AP1 | F | tggtggtgttcaagggaaaag |
|  |  | R | aggtgcaaagtctggttcagt |
|  | RAD54B | F | gccaaacactgatgatttgtgg |
|  |  | R | cctgagaagaatgcgagatagc |
|  | RECQL | F | gcgtccgtttcagctctaact |
|  |  | R | ttgccccggcatcagaatc |
|  | RFC1 | F | ttgtcatgggtcgtgatagtgg |
|  |  | R | cctggcatagtccgaatcagat |
|  | RFC3 | F | gtggacaagtatcggccctg |
|  |  | R | tgatggtccgtacactaacagat |
|  | RFC5 | F | gaagcagacgccatgactcag |
|  |  | R | gaccgaaccgaaacctcgt |
|  | RIF1 | F | agggcagactgacgcttac |
|  |  | R | gcagcactactcagctccg |
|  | SSBP1 | F | tgagtccgaaacaactaccagt |
|  |  | R | cctgatcgccacatctcattag |
|  | TDG | F | tgaagctcctaatatggcagttg |
|  |  | R | ttccactggttgttttggttct |
|  | TDP1 | F | caggaaggcgattatgggagg |
|  |  | R | ctgggaaccgcttttctgc |
|  | UPF1 | F | accgactttactcttcctagcc |
|  |  | R | aggtccttcgtgtaataggtgtc |
|  | XRCC5 | F | gtgcggtcggggaataagg |
|  |  | R | ggggattctataccaggaatgga |

**Table S2. Common gene list between KHSRP acetylation-regulated genes and DHT-induced genes in LNCaP cells.**

| Symbol | KR_vs_WT_log2FoldChange | DHT-Gene | DHT_log2FoldChange |
| --- | --- | --- | --- |
| ALKBH2 | -1.034692048 | ALKBH2 | 0.40 |
| APTX | -0.784623032 | APTX | 0.33 |
| CCNH | 0.356188608 | CCNH | 0.67 |
| CHEK1 | -0.687565841 | CHEK1 | 0.79 |
| FANCI | -1.561488453 | FANCI | 1.22 |
| FANCM | 1.742485605 | FANCM | 0.60 |
| GTF2H1 | 0.956372069 | GTF2H1 | 0.28 |
| MAD2L1 | -0.381103256 | MAD2L1 | 1.24 |
| MBD4 | -2.105604939 | MBD4 | 0.32 |
| MCM7 | -2.771812223 | MCM7 | 0.88 |
| MLH1 | 0.88303597 | MLH1 | 0.40 |
| MSH5 | -2.113645974 | MSH5 | 0.68 |
| MSH6 | 1.168448181 | MSH6 | 0.54 |
| NAE1 | 2.803651241 | NAE1 | 0.36 |
| PARP1 | -0.25392718 | PARP1 | 0.46 |
| POLA1 | 1.385551925 | POLA1 | 0.73 |
| POLD2 | -0.825922184 | POLD2 | 0.61 |
| POLR2F | 1.818277029 | POLR2F | 0.47 |
| POLR2I | -0.310788894 | POLR2I | 0.36 |
| RAD18 | -2.433783266 | RAD18 | 0.86 |
| RAD21 | -0.20923922 | RAD21 | 0.50 |
| RAD51AP1 | -3.245648393 | RAD51AP1 | 1.27 |
| RAD51C | 1.491723116 | RAD51C | 0.81 |
| RAD54B | -1.693269106 | RAD54B | 0.99 |
| RECQL | -0.242384411 | RECQL | 0.47 |
| RFC1 | -1.08034092 | RFC1 | 0.39 |
| RFC3 | -0.352778699 | RFC3 | 1.04 |
| RFC4 | 1.987856491 | RFC4 | 0.87 |
| RFC5 | -0.888966593 | RFC5 | 0.87 |
| RIF1 | -10.10402023 | RIF1 | 0.59 |
| RRM1 | 2.639894971 | RRM1 | 0.85 |
| SSBP1 | -1.278927643 | SSBP1 | 0.36 |
| TDG | -1.93971224 | TDG | 0.33 |
| TDP1 | -0.378481333 | TDP1 | 0.25 |
| UPF1 | -0.239964813 | UPF1 | 0.40 |
| WDR33 | 2.881714058 | WDR33 | 0.28 |
| XRCC5 | -0.458643659 | XRCC5 | 0.34 |
| XRCC6 | 0.731371245 | XRCC6 | 0.37 |

**Supplemental Figure legends**

**Fig.S1 Nine sites of KHSRP acetylation were identified by mass spectrometry.** HEK-293T cells were transfected with Flag-KHSRP for 36 hours before harvesting. The recombinant Flag-KHSRP protein was immunoprecipitated with the anti-Flag antibody and resolved in SDS-PAGE for Coomassie staining. The band of Flag-KHSRP was cut for the mass spectrometry analysis.

**Fig.S2 Identification of the specificity of the homemade anti-KHSRP-K205-Ac antibody.** The homemade anti-KHSRP-K205-Ac antibody successfully detected the acetylated KHSRP peptide rather than the unmodified peptide by the dot-blotting analysis.

**Fig.S3 Identification LNCaP stable cell lines.** (a) The expression levels of HA-KHSRP and endogenous KHSRP of LNCaP stable cell lines were detected by immunoblotting with indicated antibodies. (b) LNCaP cells harboring HA-KHSRP-WT were treated with DMSO or 100 nM DHT for 24 hours. Cell lysates were immunoprecipitated with anti-SIRT7 or normal rabbit IgG. The immunoprecipitates were resolved in SDS-PAGE and detected with indicated antibodies by western blotting.

**Fig.S4 KHSRP acetylation has no significant impact on miRNA biogenesis.** RNA sequencing data of differentially expressed miRNA genes between cell lines expressing KHSRP-WT and KHSRP-K205R were utilized for the volcano plotting.

**Fig.S5 The mRNA decay assay on DDR-related gene. (a-l)** LNCaP cells were treated with Actinomycin D (5μg/ml) for 2 hours before time 0. Subsequently, LNCaP-KHSRP-WT/KR cells treated with Actinomycin D for an additional 0, 8, 16, and 24 hours were collected for the qRT-PCR analysis. The expression level of each gene at each time point was presented as a remaining percentage compared with time 0. Data was plotted using GraphPad Prism by one-phase decay mode of non-linear regression (curve fitting). The data are presented as mean ± SD with t‐test analysis from three independent experiments, **p* < 0.05, ***p* < 0.01, ****p* < 0.001.
